# Peripheral immune characteristics of hepatitis B virus-related hepatocellular carcinoma

**DOI:** 10.3389/fimmu.2023.1079495

**Published:** 2023-04-03

**Authors:** Ruonan Sun, Jiawei Li, Xianyi Lin, Yidong Yang, Bing Liu, Tianbi Lan, Shuang Xiao, Anyi Deng, Zhinan Yin, Yan Xu, Zheng Xiang, Bin Wu

**Affiliations:** ^1^Department of Gastroenterology, Third Affiliated Hospital of Sun Yat-Sen University, Guangzhou, Guangdong, China; ^2^The Biomedical Translational Research Institute, Health Science Center (School of Medicine), Jinan University, Guangzhou, Guangdong, China; ^3^Department of Interventional Medicine, Zhuhai People’s Hospital (Zhuhai hospital affiliated with Jinan University), Zhuhai, Guangdong, China; ^4^Department of Hematology, Affiliated Dongguan People's Hospital, Southern Medical University (Dongguan People's Hospital), Dongguan, China; ^5^Guangzhou Purui Biotechnology Co., Ltd., Guangzhou, Guangdong, China; ^6^Department of Microbiology and Immunology, Health Science Center (School of Medicine), Jinan University, Guangzhou, Guangdong, China

**Keywords:** hepatitis B virus (HBV), hepatocellular carcinoma (HCC), cellular immunity, T cells, inhibitory receptors

## Abstract

**Background:**

Liver cancer is the sixth most common cancer worldwide and the third leading cause of cancer-related death. As a chronic liver disease, many studies have shown that the immune response plays a key role in the progression of liver cancer. Chronic hepatitis B virus (HBV) infection is one of the high-risk factors for HCC, accounting for 50%–80% of HCC cases worldwide, and little is known about the immune status of HBV associated hepatocellular carcinoma (HBV-HCC), therefore, we aimed to explore the changes in peripheral immunity in patients with HBV-HCC.

**Methods:**

In this study, patients with HBV-HCC (n=26), patients with hepatitis B-related cirrhosis (HBV-LC) (n=31) and healthy volunteers (n=49) were included. The lymphocytes and their subpopulation phenotypes in peripheral blood were characterized. In addition, we explored the effect of viral replication on peripheral immunity in patients with HCC and analyzed the circulating immunophenotypic characteristics at different stages of HCC with flow cytometry.

**Results:**

Firstly, our results showed that the percentages of total αβ T cells in the peripheral blood of HBV-HCC patients was significantly decreased compared to healthy subjects. Secondly, we found that naïve CD4^+^ T cells in HBV-HCC patients were significantly reduced, terminally differentiated CD8^+^ T cells, homing memory CD8^+^ T cells and Th2 cells were increased in peripheral circulation in HBV-HCC patients. Moreover, in the peripheral blood of HBV-HCC patients, expression of TIGIT on CD4^+^ T cells and PD-1 on the surface of Vδ 1 T cells was increased. In addition, we found that sustained viral replication resulted in up-regulation of TIM3 expression on CD4^+^ T cells, and TIM3^+^ γδ T cells increased in peripheral circulation in patients with advanced HBV-HCC.

**Conclusion:**

Our study showed that circulating lymphocytes in HBV-HCC patients exhibited features of immune exhaustion, especially in HCC patients with persistent viral replication and in patients with intermediate and advanced HBV-HCC, including decreased frequency of T cells and elevated expression of inhibitory receptors including TIGIT and TIM3 on CD4^+^ T cells and γδ T cells. Meanwhile, our research suggests that the combination of CD3^+^ T cell and CD8^+^HLADR^+^CD38^+^ T cell may be a potential diagnostic indicator for HBV-HCC. These findings could help us to better understand the immune characteristics of HBV-HCC and explore the immune mechanisms and immunotherapy strategies for HBV-HCC.

## 1 Introduction

Hepatocellular carcinoma (HCC) is one of the most common malignancies worldwide and the third leading cause of cancer-related death (1). HCC is the most common primary liver cancer, accounting for approximately 70%–80% of all primary liver cancers (2). HCC usually has a poor prognosis because it is usually multifocal at the time of discovery, prone to metastasis and a high recurrence rate after treatment (3, 4).

Chronic hepatitis B virus (HBV) infection remains a serious global public health problem, affecting 350 million people worldwide. Patients with chronic hepatitis B (CHB) are at increased risk for HBV-related cirrhosis, liver failure, and hepatocellular carcinoma (5). Although the exact mechanisms that determine the outcome of the disease remain unclear, increasing evidence suggests that the prognosis of HBV infection depends on the interaction between the virus and the host immune system. More than 80% of HCC patients in China are caused by chronic hepatitis B virus infection (6–8). Therefore, HBV infection is a key cause of HCC, and HBV-associated hepatocellular carcinoma (HBV-HCC) has always been the focus and hotspot of research. The risk of HCC development is much greater in patients with HBV-associated cirrhosis (HBV-LC) (9–11).

Studies have shown that immune system dysregulation plays an important role in the development of HCC (12, 13), and the coordinated immune response of tumor-associated immune cells, such as cytotoxic T cells, CD4^+^ T cells, Treg cells, natural killer (NK) cells has a significant impact on the development of HCC (14, 15). In particular, cytotoxic T cells play an important role in the clearance of infected or malignant hepatocytes (16). Meanwhile, recent studies have shown that B cells exhibit distinct roles in carcinogenesis and tumor progression (17–19).

Most immune checkpoint molecules include programmed cell death protein-1 (PD-1), T cell immunoglobulin domain and mucin domain-3 (TIM-3), lymphocyte-activation gene 3 (LAG-3) and T cell immune receptor with Ig and ITIM domains (TIGIT) have shown immunosuppressive activity, which can suppress T cell responses and mediate T cell exhaustion, cancer cells can evade immune surveillance by upregulating ligands for inhibitory checkpoint molecules (20–23). A new type of tumor immunotherapy, immune checkpoint inhibitors (ICIs) treat tumors by specifically blocking antibodies to block the abnormal interaction between tumor cells and T lymphocytes, thereby releasing immunosuppression and enhancing immune responses (24, 25). Immune checkpoint inhibitors are currently used as a treatment in a variety of tumors and have achieved good therapeutic effects (16, 26, 27).

A comprehensive characterization of the circulating immune landscape of HBV-HCC is still lacking, and understanding the expression of immune checkpoint molecules in different immune cells is crucial for tailoring novel immunotherapies against HBV-HCC. In addition, dynamic observation of the changes in the number of circulating lymphocytes may help to monitor the immune status of patients with high risk for HCC and HCC after treatment. Therefore, in this study, we analyzed the frequency and phenotype of various immune cells in the periphery of HBV-HCC, including T cells, B cells, NK cells, NKT cells, γδ T cells and their subsets, and the expression of immune checkpoints including PD-1, TIGIT, TIM3, and LAG3 in circulating immune cells in HBV-HCC patients. We further analyzed the relationship between immune cells and clinical parameters in patients with HBV- HCC.

## 2 Materials and methods

### 2.1 Patients

Peripheral blood samples from 26 HBV-HCC patients, 31 HBV-LC patients, and 49 healthy donors were collected in the Third Affiliated Hospital of Sun Yat-Sen University. Diagnosis of HCC and HBV-LC were confirmed according to standard guidelines (28, 29). In addition, HBV-HCC patients were defined as those who were positive for hepatitis B surface antigen (HBsAg≥6 months), had progressed to cirrhosis and met the diagnosis of HCC, and had never undergone treatment including surgery, interventional intervention, radiofrequency ablation, or chemotherapy. Clinical parameters of newly diagnosed HCC patients were collected, including gender, age, liver function test, serum alpha-fetoprotein (AFP) level and HCC stage classified by Barcelona Clinical Hepatocellular Carcinoma (BCLC) system (30). The clinical characteristics of all the enrolled populations are summarized in **Table 1**. This study complied with the Declaration of Helsinki and was approved by the Ethics Committee of the Third Affiliated Hospital of Sun Yat-sen University. All participants were provided informed consent.

**Table 1:** Clinical characteristics of enrolled subjects.

	NC(n=49)	HBV-LC(n=31)	HBV-HCC(n=26)	*p*
Gender (male/female)	27/22	19/12	22/4*	0.037
Age (year)	52.14 ± 12.73	50.90 ± 10.24	53.31 ± 10.05	0.173
AFP (<400/≥400 ng/ml)	NA	NA	17/9	
HBV-DNA (<100/≥100IU/ml)	NA	10/21	9/17	1.000
HBeAg (-/+)	49/0	24/7*	18/8*	<0.0001
WBC (*109/L)	5.94 ± 1.61	4.52 ± 2.45*	6.54 ± 3.67	0.013
RBC (*1012/L)	4.50 ± 0.41	3.86 ± 0.77*	3.94 ± 0.89*	<0.0001
Hb (g/L)	134.57 ± 12.17	110.77 ± 23.67*	118.42 ± 27.10*	<0.0001
PLT (*109/L)	218.94 ± 52.52	97.03 ± 55.89*	136.42 ± 90.00*	<0.0001
PT (s)	13.18 ± 0.64	17.35 ± 3.57*	16.34 ± 3.41*	<0.0001
INR	0.99 ± 0.06	1.40 ± 0.37*	1.30 ± 0.35*	<0.0001
ALT (U/L)	23.84 ± 20.62	81.03 ± 116.20*	79.92 ± 93.27*	0.003
AST (U/L)	21.47 ± 7.71	73.97 ± 65.62*	145.65 ± 206.88*	<0.0001
TBIL (umol/L)	10.86 ± 3.70	53.63 ± 103.01	46.47 ± 68.61*	0.005
ALB (g/L)	42.08 ± 3.23	34.34 ± 5.24*	33.81 ± 6.18*	<0.0001
BCLC stage (A/B/C/D)	NA	NA	8/2/10/6	

NC, normal controls; HBV-LC, hepatitis B virus-related liver cirrhosis; HBV-HCC, Hepatitis B virus-associated hepatocellular carcinoma; AFP, Alpha-fetoprotein; HBeAg, hepatitis B e antigen; WBC, white blood cells; RBC, red blood cells; Hb, hemoglobin; PLT, platelet; PT, prothrombin time; INR, International normalized ratio; ALT, alanine aminotransferase; AST, aspartate aminotransferase; TBIL, total bilirubin; ALB, Albumin; BCLC, Barcelona Clinic Liver Cancer; NA, not applicable.

*p value < 0.05 vs normal controls.

### 2.2 Isolation of peripheral blood mononuclear cells and multi-parametric flow cytometric analysis

In this study, 5 ml of peripheral blood was collected from all participants and placed in heparin sodium anticoagulant blood collection tubes. Peripheral blood mononuclear cells (PBMCs) were isolated from peripheral blood by Ficoll density gradient centrifugation (31). Flow cytometric analysis of PBMCs from patients and healthy controls was performed and different surface markers were stained with mouse anti-human fluorescein conjugated to different monoclonal antibodies (**Supplementary Table 1**). Samples were incubated with antibodies for 30 min at 4°C, then washed 2 times with PBS, cells were resuspended in 200ul PBS. Finally, BD FACSLyric was used to collect samples and obtain experimental data, followed by data analysis using FlowJo software (version 10).

### 2.3 Statistical analysis

Data are presented as mean ± SD or median and quartiles (25th and 75th) depending on the data distribution. T-test and ANOVA were used for normally distributed data, and the nonparametric tests Mann-Whitney U test and Kruskal-Wallis H test were used for non-normally distributed data. Pearson’s and Spearman’s correlation coefficients were used to assess the correlation between normally distributed and non-normally distributed data, respectively. All data were analyzed using SPSS software version 25.0 and GraphPad Prism version 9.0. P values < 0.05 were considered statistically significant.

## 3 Results

### 3.1 General immune characteristics in the peripheral circulation of HBV-HCC patients

**Figure 1:**
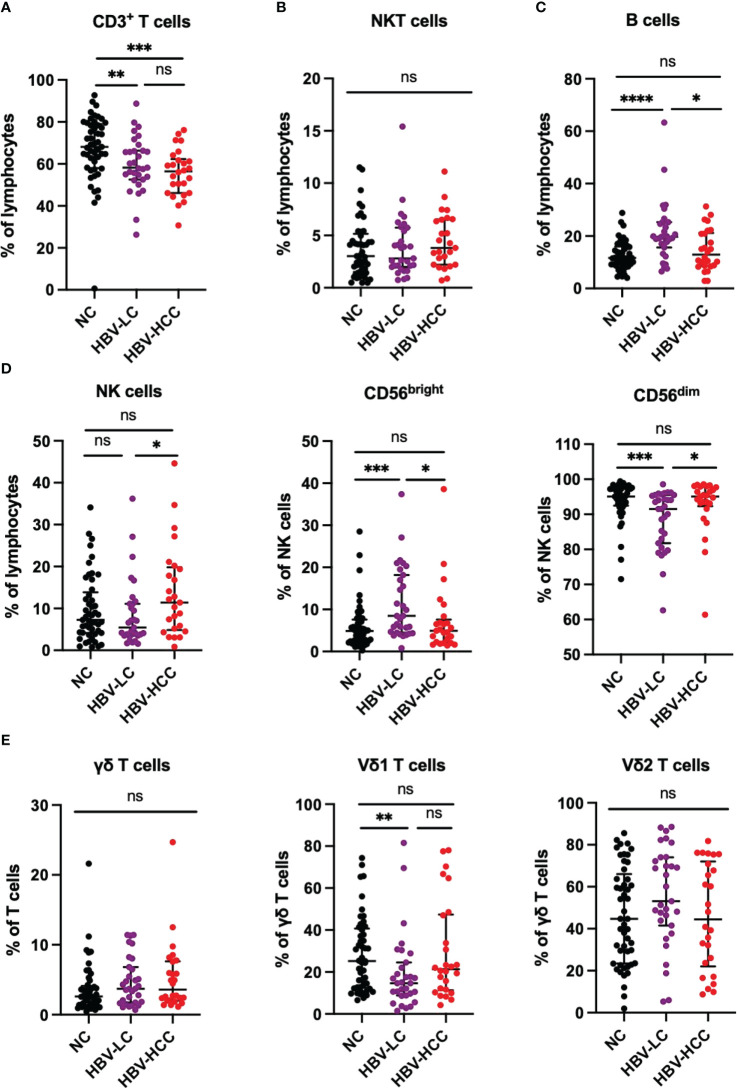
Peripheral circulating lymphocytes from NC (n=49), HBV-LC (n=31) and HBV-HCC (n = 26) patients were analyzed by multiparameter flow cytometry. **(A)** Frequency of CD3^+^ T cells in peripheral blood lymphocytes. **(B)** NKT cell (CD3^+^CD56^+^) frequency in peripheral blood lymphocytes. **(C)** Frequency of B cells in peripheral blood lymphocytes. **(D)** NK cells (CD3^-^CD56^+^) were further classified into CD56^bright^ NK cells and CD56^dim^ NK cells according to CD56 expression. **(E)** Circulating γδ T cells (CD3 ^+^ γδ TCR ^+^) and their subsets Vδ1 and Vδ2 frequencies. The ANOVA or Kruskal-Wallis test was used to determine statistical differences between the three groups. *p<0.05, **p<0.01, ***p<0.001, ****p<0.0001. ns, not significant.

Firstly, we analyzed the peripheral blood lymphocyte subsets of NC, HBV-LC and HBC-HCC patients to understand the general distribution of PB T cells, B cells, NK cells, NKT cells, γδ T cells. We found that the frequency of CD3^+^ T cells in peripheral blood lymphocytes of patients with HBV-HCC was significantly lower than that of healthy volunteers (*p*<0.0001), and the frequency of CD3^+^ T cells in peripheral blood of patients with HBV-LC was significantly lower than that of healthy volunteers (*p*<0.01), however, there was no significant difference between HBV-HCC patients and HBV-LC patients (**Figure 1A**; **Supplementary Figure 1A**). The frequency of circulating B cells in HBV-HCC patients was significantly lower than that in HBV-LC patients (*p*<0.05), and the circulating B cells in HBV-LC patients were significantly higher than those in healthy donors (*p*<0.0001), but there was no statistical difference between HBV-HCC patients and healthy donors (**Figure 1C**). Subsequently, we compared the frequency of NK cells in the peripheral blood of the three groups and found that the frequency of NK cells in HBV-HCC patients was significantly higher than that in HBV-LC patients (*p*<0.05), mainly CD56^bright^ NK cells (*p*<0.05), while there was no statistical difference between HBV-HCC patients and healthy volunteers (**Figure 1D**). In addition, we also compared the frequency of circulating NKT cells and γδ T cells in HBV-HCC patients, HBV-LC patients and healthy volunteers, and the results showed no significant difference between the three groups (**Figures 1B, E**).

### 3.2 The immune phenotype of αβ T cells in peripheral circulation of patients with HBV-HCC

**Figure 2:**
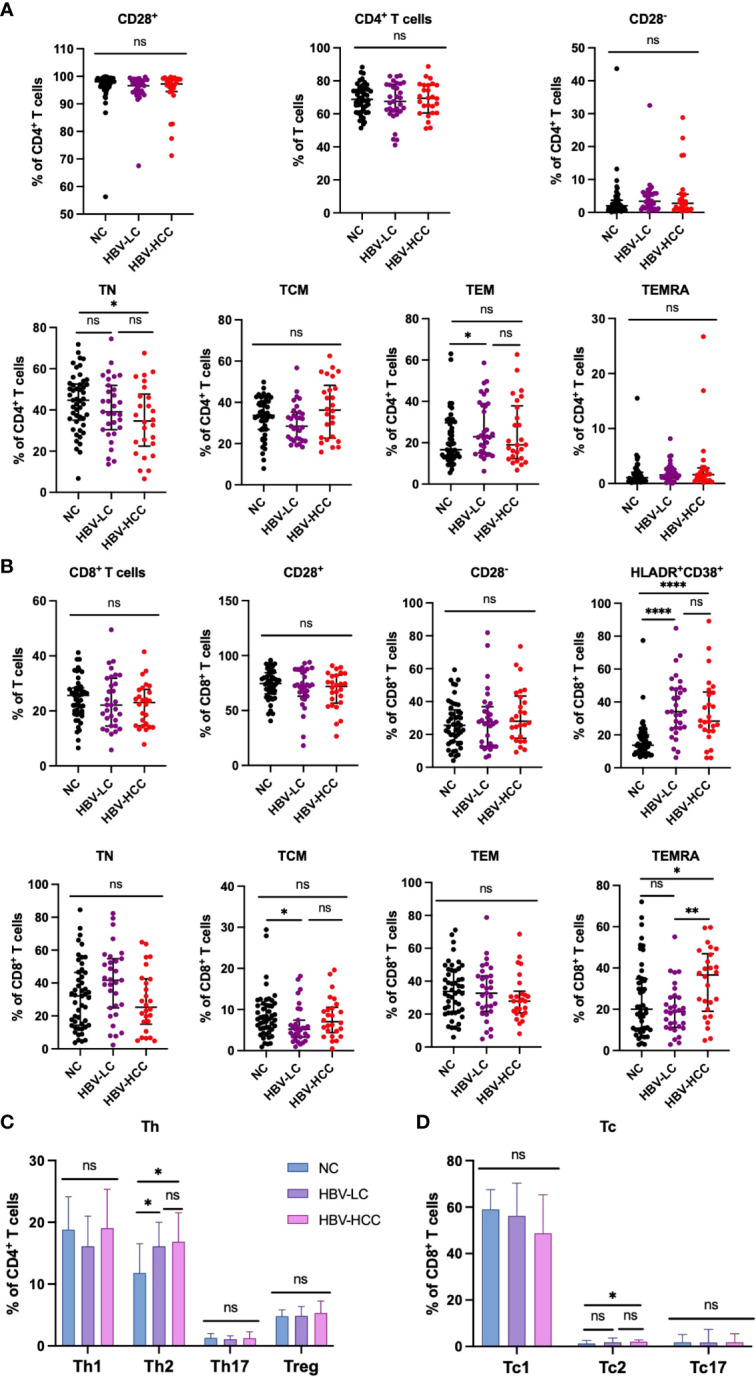
Frequency of circulating αβ T cells and subsets in healthy volunteers、HBV-LC and HBV-HCC patients. **(A)** According to the expression of co-stimulatory molecule CD28, CD4^+^ T cells can be divided into functional cells (CD28 ^+^) and exhausted cells (CD28^-^), proportion of TN, TCM, TEM and TEMRA subsets of CD4^+^ T cells. **(B)** According to the expression of co-stimulatory molecule CD28, CD8^+^ T cells can be divided into functional cells (CD28^+^) and exhausted cells (CD28^-^), proportion of TN, TCM, TEM and TEMRA subsets of CD8^+^ T cells. Marker of activation HLADR and CD38 were stained to determine the homing memory CD8^+^ T cells. **(C)** The frequency of Th1, Th2, Th17, Treg cells in CD4^+^ T cells. **(D)** The frequency of Tc1, Tc2 and Tc17 cells in CD8^+^ T cells. The ANOVA or Kruskal-Wallis test was used to determine statistical differences between the three groups. *p<0.05, **p<0.01, ****p<0.0001. ns, not significant.

Next, we performed immune phenotype of αβ T cells. According to the expression of CD28, CD38, CCR7, CD45RA and HLADR, which reflect the characteristics of cell activation, exhaustion, memory and homing, the CD4^+^ T cells and CD8^+^ T cells were divided into functional T cells (CD28^+^), exhausted T cells (CD28^-^), naïve T cells (TN; CD45RA^+^CCR7^+^), central memory T cells (TCM; CD45RA^-^CCR7^+^), effector memory T cells (TEM; CD45RA^-^CCR7^-^), terminally differentiated effector T cells (TEMRA; CD45RA^+^ CCR7^-^), and homing memory T cells (HLADR^+^CD38^+^). We found that naïve CD4^+^ T cells in HBV-HCC patients were significantly lower than healthy volunteers (*p*<0.05), and naïve CD4^+^ T cells in HBV-HCC patients were lower than those in HBV-LC patients (**Figure 2A**). We did not find significant differences in CD28^+^, CD28^-^, TCM, TEM and TEMRA cells in CD4^+^ T cells between HBV-HCC patients and healthy volunteers (**Figure 2A**). Similarly, cluster analysis of CD8^+^ T cells showed that the frequency of terminally differentiated effector CD8^+^ T (TEMRA) cells in the circulation of HBV-HCC patients was significantly higher than that of healthy volunteers (*P <*0.05) and HBV-LC patients (*p <*0.01) (**Figure 2B**; **Supplementary Figure 1B**). The expressions of CD38 and HLADR on CD8^+^ T cells in peripheral blood of HBV-HCC patients were significantly higher than those of healthy volunteers (*p <*0.0001), and the expressions of CD38 and HLADR on CD8^+^ T cells in peripheral blood of HBV-LC patients were significantly higher than those of healthy volunteers (*p <*0.0001), but there was no statistical difference between HBV-HCC and HBV-LC patients (**Figure 2B**). Next, we detected Treg cells, Th subsets in CD4^+^ T cells and Tc subsets in CD8^+^ T cells, the results showed the frequency of Th2 cells in the circulation of patients with HBV-HCC and HBV-LC was significantly higher than that in healthy volunteers (*p*<0.05, *p*<0.05), while there was no significant difference in the frequency of Th2 cells between patients with HBV-HCC and patients with HBV-LC (**Figure 2C**). The frequency of circulating Tc2 cells in HBV-HCC patients was higher than that in healthy volunteers (*p*<0.05), and there was no statistical difference between HBV-HCC patients and HBV-LC patients (**Figure 2D**). There was no significant difference in the frequency of Treg cells among the three groups (**Figure 2C**). In addition, we measured the frequency of Tfh subsets and there had no statistical differences were found between the groups (**Supplementary Figure 2A**), we also measured the B cells and found that among B cell subsets, the frequency of naïve B cells in HBV-HCC patients was significantly higher than that in healthy volunteers (*p*<0.0001), and the frequency of MZ B cells was significantly lower than that in healthy volunteers (*p*<0.0001), as well as the frequency of class-switched B cells and plasma cells (*p*<0.0001) (**Supplementary Figure 2B**).

### 3.3 The expression of inhibitory receptors on αβ T cells and γδ T cells in HBV-HCC patients

**Figure 3:**
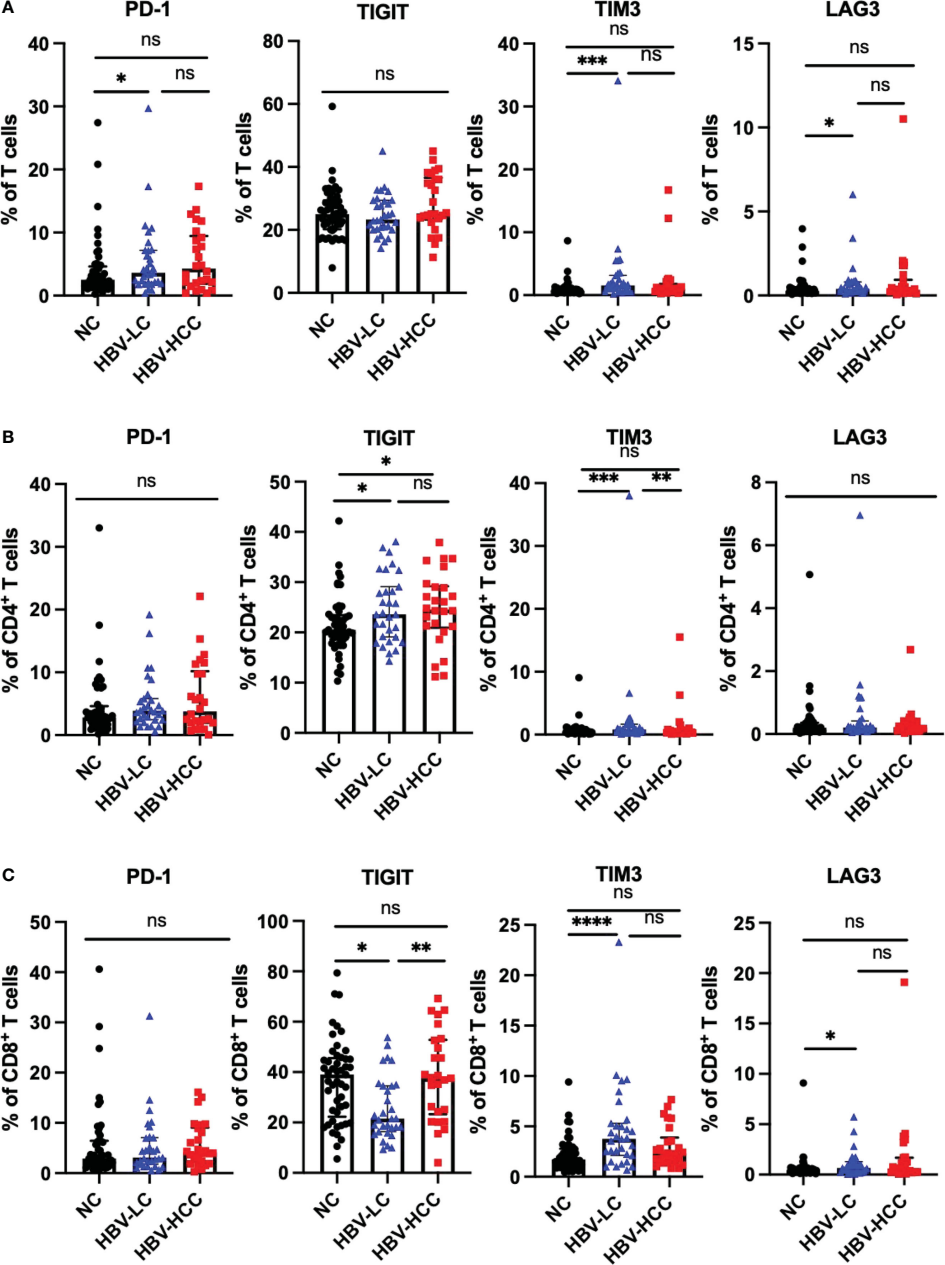
Expression of inhibitory receptors on αβ T cells. **(A)** Expression of PD-1, TIGIT, TIM3 and LAG3 on CD3^+^ T cells. **(B)** Expression of PD-1, TIGIT, TIM3 and LAG3 on CD4^+^ T cells. **(C)** Expression of PD-1, TIGIT, TIM3 and LAG3 on CD8^+^ T cells. *p<0.05, **p<0.01, ***p<0.001, ****p<0.0001. ns, not significant.

**Figure 4:**
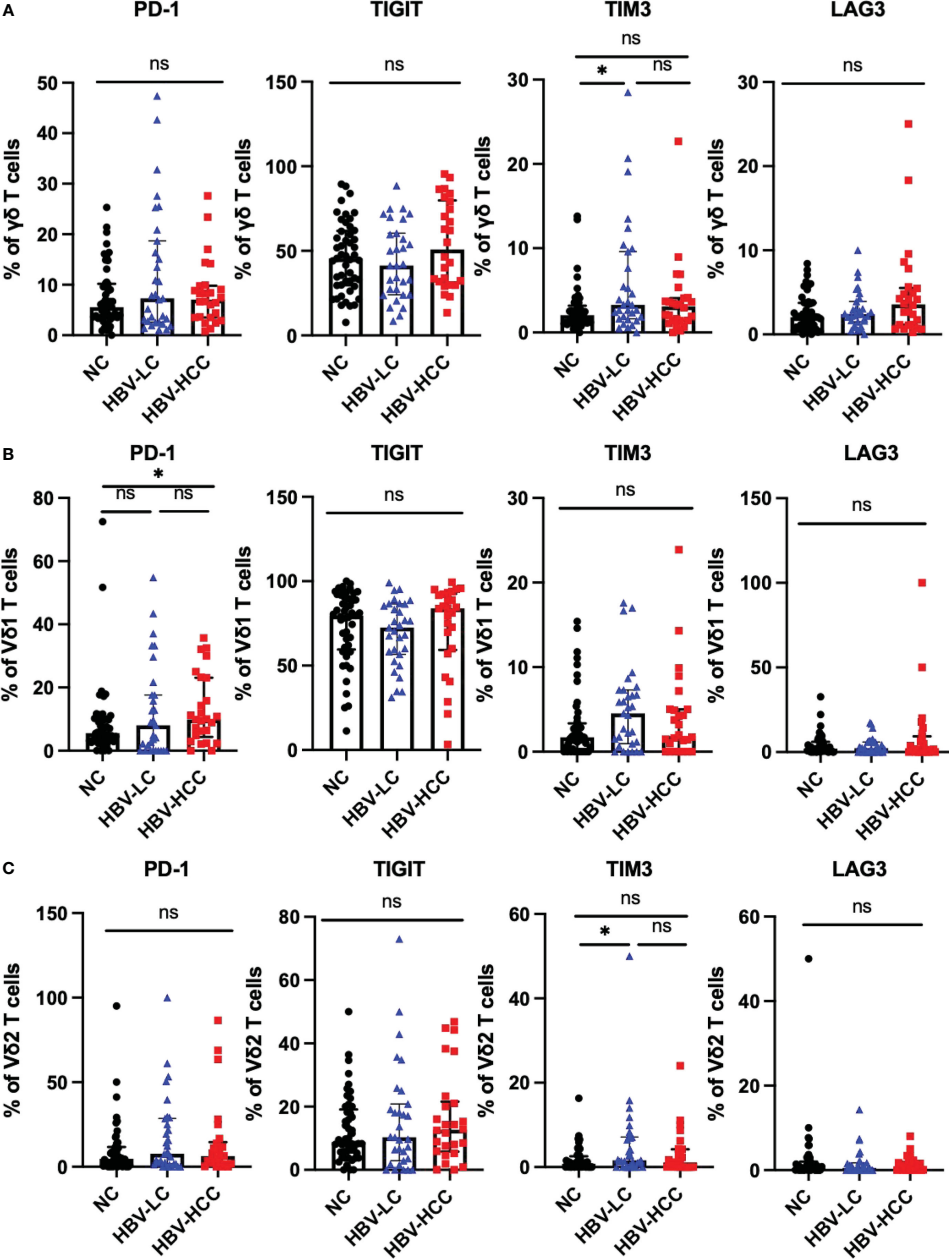
Expression of inhibitory receptors on γδ T cells. **(A)** Expression of PD-1, TIGIT, TIM3 and LAG3 on the surface of γδ T cells. **(B)** The expression of PD-1, TIGIT, TIM3 and LAG3 on the surface of Vδ1 cells. **(C)** The expression of PD-1, TIGIT, TIM3 and LAG3 on the surface of Vδ2 cells. *p<0.05.

We next assessed the expression of inhibitory receptors including PD-1, TIGIT, TIM3, and LAG3 on αβ T cells and γδ T cells. The results showed that compared with healthy volunteers, the expression of TIGIT on circulating CD4^+^ T cells in HBV-HCC patients was increased (*p*<0.05) (**Figure 3B**). We did not find significant differences in the expression of inhibitory receptors on αβ T cells and CD8^+^ T cells between HBV-HCC patients and healthy volunteers (**Figures 3A, C**). The expression of PD-1 on the surface of Vδ1 T cells in HBV-HCC patients was higher than that in healthy volunteers. However, there was no significant difference compared with HBV-LC patients (**Figure 4B**). We did not find differences in the expression of inhibitory receptors on γδ T cells and Vδ2 T cells in the peripheral circulation of HBV-HCC patients and healthy volunteers (**Figures 4A, C**). In addition, killer cell immunoglobulin-like receptors (KIRs) are key regulators of NK cell function, with activating or inhibitory functions, we measured the expression of KIR on NK cells and found no significant differences among groups (**Supplementary Figures 2C, D**).

### 3.4 The expression of activating receptors on NK cells and γδ T cells in HBV-HCC patients

**Figure 5:**
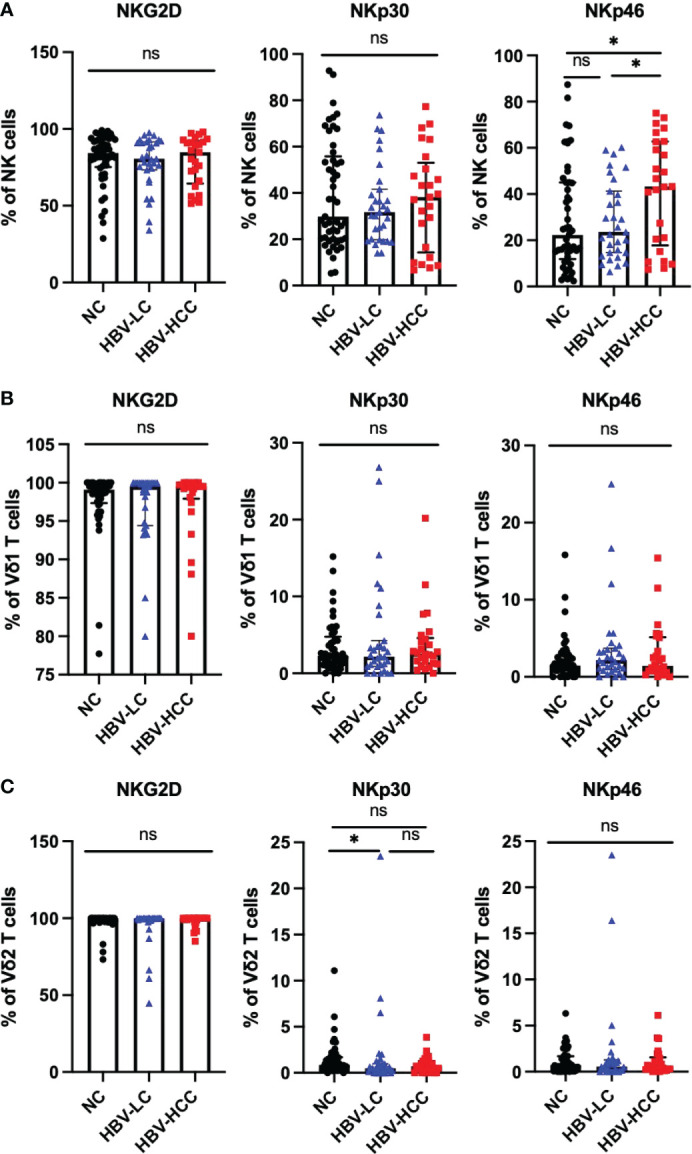
Expression of activating receptors on NK cells and γδ T cells. **(A)** Expression of NKG2D, NKP30, NKP46 on the surface of NK cells. **(B)** The expression of NKG2D, NKp30, NKp46 on the surface of Vδ1 cells. **(C)** The expression of NKG2D, NKP30, NKP46 on the surface of Vδ2 cells. *p<0.05. ns, not significant.

NKG2D, NKp30, and NKp46 are important activating receptors on immune cells, which mediate the activation of immune cells, and their expression levels are positively correlated with the anti-tumor ability of immune cells. We analyzed the expression of activating receptors on NK cells, γδ T cells, it was found that compared with healthy volunteers and HBV-LC patients, the expression of NKp46 was up-regulated on circulating NK cells in HBV-HCC patients (**Figure 5A**). There was no significant difference in the expression of activating receptors on the surface of Vδ1 T cells and Vδ2 T cells between HBV-HCC patients and healthy controls (**Figures 5B, C**).

### 3.5 The correlation between clinical characteristics and immune cell phenotypes in peripheral blood of HBV-HCC patients

**Figure 6:**
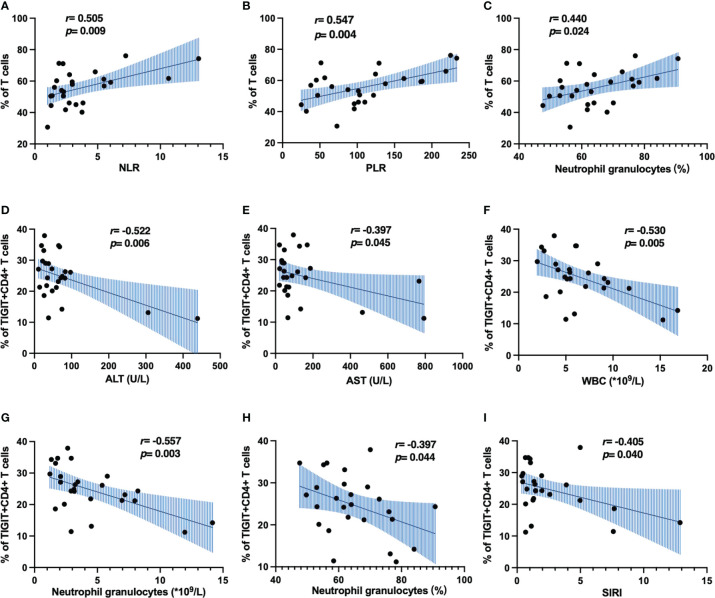
Correlation analysis of immune cell phenotype and clinical indicators in patients with HBV-HCC. **(A–C)** The correlation of CD3^+^ T cells and inflammatory markers. **(D–I)** The correlation of TIGIT^+^CD4^+^ T cells and inflammatory markers.

Next, we analyzed the correlation between clinical indicators and immune cell phenotype in patients with HBV-HCC. Inflammatory response is closely related to the occurrence and development of tumors. Neutrophil-to-lymphocyte ratio (NLR), platelet-to-lymphocyte ratio (PLR) and systemic inflammatory response index (SIRI) are widely used in the evaluation of inflammatory diseases (32), which are closely related to the immune response of the body, especially to the progression and prognosis of HCC (33). We found that the frequency of αβ T cells was positively correlated with NLR, PLR and percentage of neutrophils (**Figures 6A–C**), The frequency of TIGIT^+^CD4^+^ T cells was negatively correlated with WBC, neutrophil counts, percentage of neutrophils, SIRI, ALT and AST (**Figures 6D–I**). Meanwhile, we also found the frequency of MZ B cells was positively correlated with NLR, PLR and percentage of neutrophils (**Supplementary Figures 2E–G**). These results suggest that αβ T cells, MZ B cells and TIGIT^+^CD4^+^ T cells may play different roles in the development of HBV-HCC.

### 3.6 Effect of viral replication on peripheral immunophenotype in patients with HBV-HCC

**Figure 7:**
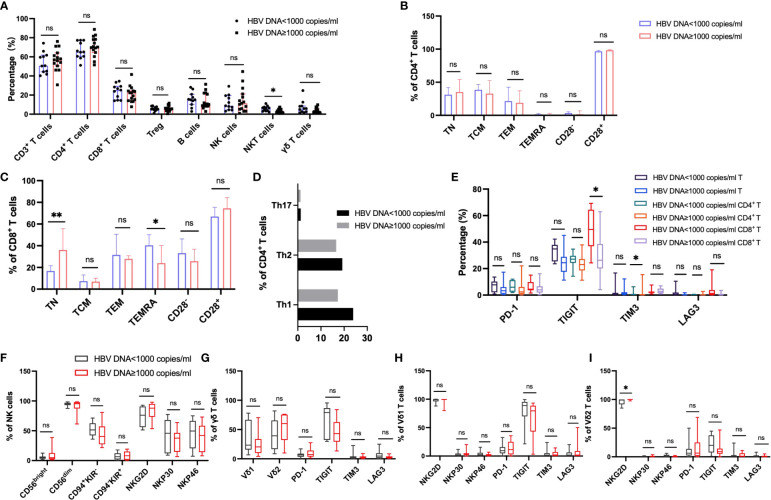
Circulating lymphocytes and their immunophenotypic characteristics in the patients with HBV DNA<1000 copies/ml and patients with HBV DNA≥1000 copies/ml. **(A)** The frequencies of αβ T, B, NK, NKT and γδ T cells in the two groups. **(B, C)** The expression of CD28, CD45RA and CCR7 on the surface of CD4^+^ T cells and CD8^+^ T cells in the two groups. **(D)** The frequency of CD4^+^ T cell subsets in the two groups. **(E)** Differences in the expression of inhibitory receptors on the surface of peripheral blood CD3^+^ T cells, CD4^+^ T cells, and CD8^+^ T cells between the two groups. **(F)** The expression of cytotoxic and activating receptors of circulating NK cells in two groups. **(G–I)** Differences in the expression of activated receptors and inhibitory receptors on the surface of γδ T cells and its subsets Vδ1 and Vδ2 cells between the two groups. T test or Mann-Whitney U test was used to determine statistical differences between the two groups. *p<0.05, **p<0.01. ns, not significant.

In order to explore the effect of HBV viral load on peripheral immunity of HCC, we divided HBV-HCC patients into two groups according to HBV DNA load. Peripheral immune cell phenotypes were analyzed in patients with HBV DNA <1000 copies/ml which was referred to as the non-viral replication group and patients with HBV DNA ≥1000 copies/ml which was referred to as the viral replication group. We found that the frequency of NKT cells in the non-viral replication group was higher than that in the virus replication group (*p*<0.05), and there was no significant difference in the frequency of other immune cells and subsets between the two groups (**Figure 7A**). According to the expression of CD45RA and CCR7, CD4^+^ T cells and CD8^+^ T cell subsets were analyzed, and it was found that the frequency of TN in CD8^+^ T cells in the viral replication group was significantly higher than that in the non-viral replication group, and the frequency of TEMRA was significantly lower than that in the non-viral replication group, and there was no significant difference in the frequency of CD4^+^ T cell subsets between the two groups (**Figures 7B–D**). The analysis of activating receptors and inhibitory receptors showed that the expression of TIGIT on CD8^+^ T cells in the non-viral replication group was significantly higher than that in the viral replication group, and the expression of TIM3 on CD4^+^ T cells was lower than that in the viral replication group (**Figure 7E**). The expression of NKG2D in Vδ2 T cells in the viral replication group was higher than that in the non-viral replication group, and there was no significant difference in activating receptors and inhibitory receptors on NK cells, γδ T cells and Vδ1 T cells between the two groups (**Figures 7F–I**). In addition, we did not find significant differences in subsets of Tfh and B cells between groups (**Supplementary Figures 2H, I**)

### 3.7 Effect of disease progression on peripheral immunophenotype in patients with HBV-HCC

**Figure 8:**
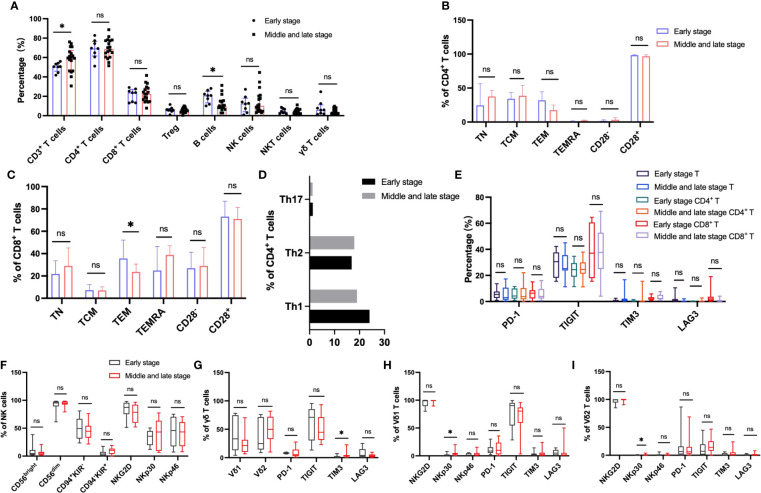
Circulating lymphocytes and their immunophenotypic characteristics in patients with early and advanced HBV-HCC. **(A)** The frequencies of αβ T, B, NK, NKT and γδ T cells in the two groups. **(B, C)** The expression of CD28, CD45RA and CCR7 on the surface of CD4^+^ T cells and CD8^+^ T cells in the two groups. **(D)** The frequency of CD4^+^ T cell subsets in the two groups. **(E)** Differences in the expression of inhibitory receptors on the surface of peripheral blood CD3^+^ T cells, CD4^+^ T cells, and CD8^+^ T cells between the two groups. **(F)** The expression of cytotoxic and activating receptors of circulating NK cells in two groups. **(G–I)** Differences in the expression of activated receptors and inhibitory receptors on the surface of γδ T cells and its subsets Vδ1 and Vδ2 cells between the two groups. T test or Mann-Whitney U test was used to determine statistical differences between the two groups. *p<0.05. ns, not significant.

In order to explore the immune characteristics of HBV-HCC in different stages, we divided HBV-HCC into stage 0 (the earliest stage), stage A (early stage), stage B (middle stage), stage C (late stage) and stage D (end stage) according to BCLC system. We classified patients with stage 0 and A as early HCC group, and patients with stage B-D as middle and late HCC group, and analyzed the differences in circulating immunity between the two groups. The results showed that compared with the HBV-HCC patients in early stage, the frequency of circulating αβ T cells was significantly higher in patients with advanced HBV-HCC, there were no significant differences in the frequencies of other immune cells (**Figure 8A**). According to the expression of CD45RA and CCR7, the analysis of CD4^+^ T cells and CD8^+^ T cell subsets showed that the frequency of TEM in CD8^+^ T cells of patients with advanced HCC was significantly lower than that of patients with early HCC, and there was no significant difference in the frequency of other CD4^+^ T cell subsets between the two groups (**Figures 8B–D**). Analysis of the inhibitory receptors of αβ T cells, CD4^+^ T cells and CD8^+^ T cells in the two groups showed that the expressions of early HCC and advanced HCC patients were similar (**Figure 8E**). The cell surface activating receptor NKp30 was up-regulated in Vδ1 T cells and Vδ2 T cells of patients with advanced HCC, and TIM3 was up-regulated in circulating γδ T cells of patients with advanced HCC. The expression of inhibitory and activating receptors and inhibitory receptors on the surface of circulating NK cells was similar between the two groups (**Figures 8F–I**). Meanwhile, the frequency of B cells was significantly lower in patients with advanced HBV-HCC, among which naïve B cells were significantly lower than those in patients with early HCC, and class-switched B cells and plasma cells were significantly higher than those in patients with early HCC (**Supplementary Figures 2J, K**).

### 3.8 Peripheral circulating lymphocytes may predict the occurrence of HBV‑HCC

**Figure 9:**
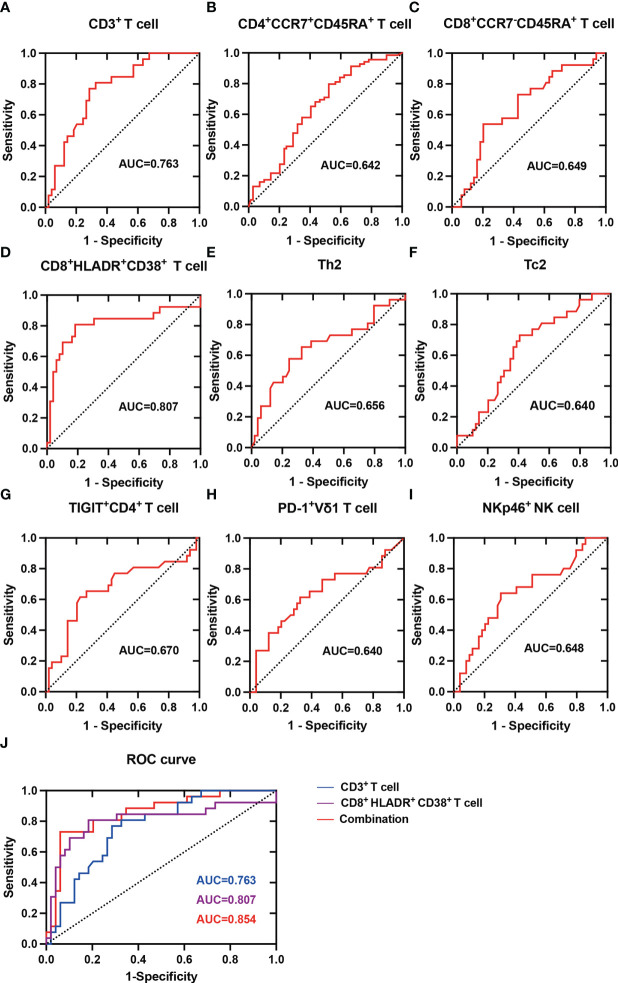
ROC analysis of Peripheral circulating lymphocytes in predicting HBV-HCC. **(A)** ROC curve for the frequency of CD3^+^ T cell as a predictor of HBV-HCC. **(B)** ROC curve for the frequency of CD4^+^CCR7^+^CD45RA^+^ T cell as a predictor of HBV-HCC. **(C)** ROC curve for the frequency of CD8^+^CCR7^-^CD45RA^+^ T cell as a predictor of HBV-HCC. **(D)** ROC curve for the frequency of CD8^+^HLADR^+^CD38^+^ T cell as a predictor of HBV-HCC. **(E)** ROC curve for the frequency of Th2 as a predictor of HBV-HCC. **(F)** ROC curve for the frequency of Tc2 as a predictor of HBV-HCC. **(G)** ROC curve for the frequency of CD4^+^TIGIT^+^ T cell as a predictor of HBV-HCC. **(H)** ROC curve for the frequency of PD-1^+^ Vδ1 T cell as a predictor of HBV-HCC. **(I)** ROC curve for the frequency of NKp46^+^ NK cell as a predictor of HBV-HCC. **(J)** ROC curve for the frequency of CD3^+^ T cell, CD8^+^HLADR^+^CD38^+^ T cell and the combination of both as a predictor of HBV-HCC.

To determine whether peripheral circulating lymphocytes are associated with diagnosis of HBV-HCC, we plotted receiver-operating characteristic (ROC) curve for the frequency of lymphocytes which was different between healthy controls and HBV-HCC patients as a predictor of HBV-HCC. The area under the receiver-operating characteristic (AUROC) curve for CD3^+^ T cell was 0.763 (95% CI 0.655-0.871, *P*=0.0002) (**Figure 9A**), the AUROC for CD4^+^CCR7^+^CD45RA^+^ T cell was 0.642 (95% CI 0.550-0.735, *P*=0.004) (**Figure 9B**), the AUROC for CD8^+^CCR7^-^CD45RA^+^ T cell was 0.649 (95% CI 0.519-0.778, *P*=0.035) (**Figure 9C**), the AUROC for CD8^+^HLADR^+^CD38^+^ T cell was 0.807 (95% CI 0.684-0.930, *P***<**0.0001) (**Figure 9D**), the AUROC for Th2 was 0.656 (95% CI 0.519-0.794, *P*=0.027) (**Figure 9E**), the AUROC for Tc2 was 0.640 (95% CI 0.513-0.768, *P*=0.047) (**Figure 9F**), the AUROC for CD4^+^TIGIT^+^ T cell was 0.670 (95% CI 0.531-0.810, *P*=0.016) (**Figure 9G**), the AUROC for PD-1^+^ Vδ1 T cell was 0.640 (95% CI 0.500-0.781, *P*=0.047) (**Figure 9H**), the AUROC for NKp46^+^ NK cell was 0.648 (95% CI 0.514-0.782, *P*=0.038) (**Figure 9I**). These results indicated that the combination of CD3^+^ T cell and CD8^+^HLADR^+^CD38^+^ T cell could be used as a potential indicator to predict HBV-HCC. The ROC curve showed that the optimal cut-off value of CD3^+^ T cell was 62.25%, and the sensitivity and specificity to predict HBV-HCC were 76.9% and 71.4%, and the optimal cut-off value of CD8^+^HLADR^+^CD38^+^ T cell was 21.90%, and the sensitivity and specificity to predict HBV-HCC were 80.7% and 81.6%. In addition, we made the ROC curve of the combination of CD3^+^ T cell and CD8^+^HLADR^+^CD38^+^ T cell, and the AUROC was 0.854 (**Figure 9J**). It indicated that the diagnostic efficacy of combination of the two indexes may be better. Furthermore, to determine whether peripheral circulating lymphocytes are associated with prognosis in HBV-HCC, we followed up the HBV-HCC patients for a median of 46 weeks (28–60 weeks). All HBV-HCC patients were divided into two groups according to the the median level of peripheral circulating lymphocytes which was different between healthy controls and HBV-HCC patients. By Kaplan–Meier survival analysis, we found that there had no difference in the overall survival rate between the two groups (**Supplementary Figures 3A–I**).

## 4 Discussion

T cell exhaustion is a state of T cell dysfunction caused by persistent antigenic stimulation during chronic infection and cancer, leading to tumor immune escape by hindering tumor clearance (20, 34). We found that αβ T cells in the peripheral circulation of patients with HBV-HCC were significantly decreased. And the increased expression of inhibitory receptors was a key feature of this phenomenon, consistent with the increased expression of TIGIT on CD4^+^ T cells of HBV-HCC patients in our study, this provides a potential rationale for developing therapeutic modalities targeting TIGIT to prevent HCC progression and promote survival. Moreover, our results showed the correlation between TIGIT^+^CD4^+^ T cells and clinical inflammatory indicators, suggesting that TIGIT^+^CD4^+^ T cells may be used as an indicator to predict the occurrence and development of HBV-HCC. At the same time, we found that compared with HBV-LC patients, the expression of TIM3 on peripheral blood CD4^+^ T cells and TIGIT on CD8^+^ T cells in HBV-HCC patients increased, which was consistent with previous research results (35). This suggests that targeted immunotherapy based on TIGIT on the surface of CD8^+^ T cells may be an effective treatment for HCC. Substantial evidence suggests that dysfunction of the host antiviral immune response is the main cause of persistent HBV replication, and we found that TIM3 expression on CD4^+^ T cells was up-regulated in HCC patients with high HBV DNA load, suggesting that TIM3 may serve as a potential therapeutic target for HCC patients with high viral burden. Naïve CD8^+^ T cells are elevated in the viral replication group, which appeared to be associated with continued exposure to high levels of viral antigens. A similar explanation could be provided by a mouse model of previous chronic polyomavirus infection, in which a sustained recruitment of naïve cells was demonstrated in the face of sustained antigenic stimulation (36).

The αβ T cells compartment can be divided into distinct subpopulations based on their ability to maintain long-term immune memory, and the naïve CD4^+^ T cell compartment has long been thought to consist of a homogeneous population of cells that have not experienced antigens (37). In this study, we observed a lower proportion of naïve CD4^+^ T cells in HBV-HCC patients and HBV-LC compared with healthy controls, which may be related to HBV infection and liver disease status, and persistent antigenic stimulation leads to differentiation of CD4^+^ T cells, which may be related to the occurrence and development of tumors. We found that among CD8^+^ T cell subsets, Compared with healthy controls and HBV-LC patients, TEMRA was significantly increased in PBMCs of HBV-HCC patients, which is consistent with previous findings (38), which may be related to the decreased recruitment of TEMRA to tumor sites. The co-expression of human leukocyte antigen DR (HLA-DR) and CD38 is related to the activation of CD8^+^ T cells, and the circulating activated CD8^+^ T cells (CD38^+^HLADR^+^) in patients with HBV-HCC are significantly higher than those in healthy controls, indicating that HBV-HCC patients have an effective adaptive immune response. CD4^+^ helper T (Th) cells are key modulators of tumor immunity. Based on cytokine production and immune function, cells are divided into distinct subpopulations: Th1 cells, Th2 cells, and Th17 cells, etc (39). Th1 cells produce characteristic cytokines interleukin (IL)-2, interferon (IFN)-γ, and tumor necrosis factor (TNF)-α, which mainly mediate antitumor immunity and are associated with favorable prognosis in HCC patients. Meanwhile, Th2 cells secrete IL-4 and IL-10, which promote tumor growth or metastasis through immunosuppression (40, 41). Our findings showed an increase in Th2 cells in the peripheral circulation of HBV-HCC patients. Similarly, the study by Foerster et al. showed an increased frequency of Th2 cells in HCC tissues compared to normal tissues (42), suggesting that developing novel strategy to alleviate Th2 cell infiltration may be helpful for the treatment of HBV-HCC.

Normal B cell development and survival can promote an efficient immune response to pathogen clearance, whereas abnormal B cell differentiation and activation can lead to disruption of B-cell homeostasis (43). Recent evidence suggests that B cells exhibit dual biological effects in the development and progression of several cancers (44). Studies have shown that MZ B cells may be able to mimic the antigen-presenting activity of traditional dendritic cells (cDCs) and present antigens to CD4^+^ T cells to modulate their immune responses (45). In the present study, we found an increase in naïve B cells and a significant decrease in the frequency of MZ B cells, class-switched B cells and plasma cells in HBV-HCC patients, consistent with previous reports (43, 46), which reflects immune deficiency in HBV-HCC patients. And the results were contrary in advanced HBV-HCC. At present, the role of B lymphocytes in the development of HBV-HCC is still controversial and needs further study.

NK and γδ T cells provide the first line of defense against virus-infected cells and tumors, and their function is regulated by a balance between inhibitory and activating receptors. NK cell function is regulated by HLA class I-specific inhibitory receptors (KIR and CD94) and many non-HLA-specific receptors including activation markers NKp30, NKp44, NKp46 and NKG2D (47). Previous studies have shown that the expression of NKp46 is positively correlated with cytotoxicity (48, 49) and plays an important role in eliminating virus-infected cells by recognizing viral proteins (50, 51). Our study showed that the frequency of NK cells in peripheral blood of HBV-HCC patients is higher than that of HBV-LC patients, especially CD56^dim^ NK cells, which is different from previous research results (52), which may be related to the small sample size in this study. At the same time, the NKp46 of NK cells in the peripheral blood of HBV-HCC patients was significantly up-regulated, which was inconsistent with the reported decrease in the expression of NKp46 in peripheral NK cells of HCC (47), which may be related to chronic HBV infection in the HCC patients (53, 54). Two main subsets of γδ T cells in human peripheral blood, Vδ1 and Vγ9Vδ2, we found that the expression of PD-1 on Vδ1 cells was increased in HBV-HCC patients, and we found that TIM3^+^ γδ T in peripheral blood of patients with advanced HBV-HCC was higher than that in early stage, suggesting that the expression of TIM3 on γδ T cells may be related to disease progression. Virus replication leads to the increased expression of NKG2D on Vδ2 T cells, which may be related to the enhanced cell activation and killing ability caused by continuous virus stimulation.

To determine whether peripheral circulating lymphocytes are associated with diagnosis of HBV-HCC, we plotted ROC curve for the frequency of lymphocytes which was different between healthy controls and HBV-HCC patients, the results demonstrated that the combination of CD3^+^ T cell and CD8^+^HLADR^+^CD38^+^ T cell may be a potential diagnostic indicator for HBV-HCC. Immunotherapy is a very promising approach to treat HCC patients, such as PD-1 inhibitors, chimeric antigen receptor T-cell therapy, and adoptive immunotherapy based on NK or γδ T cells, especially for patients who are not candidates for surgery, interventional or patients with recurrence. Monitoring immune status is crucial for personalized immunomodulatory therapy to achieve better therapeutic effects. Peripheral blood immune cell detection is non-invasive, convenient, reproducible, and relatively easy to apply in clinical practice. In view of the different responses of HCC patients to treatment, especially HCC patients with HBV infection, it is necessary to find new immunotherapy targets, and at the same time, we can formulate targeted treatment strategies according to the immune damage of each patient. At present, on the basis of the existing traditional treatment methods for HCC, it is still a difficult problem to determine the optimal regimen and timing of combined immunotherapy. There are still some limitations in our study. Firstly, in this study, we carried out a detailed analysis of the peripheral immune characteristics of HBV-HCC patients, but lacked the exploration of the liver immune microenvironment in HBV-HCC patients, a recent study showed that immune cell subsets have an immunosuppressive gradient in the tumor microenvironment of circulating blood, non-tumor tissues and liver cancer tissues (55). Further studies are needed to elucidate the interactions and mechanisms between tumors and the immune system. Secondly, due to the limited sample size, some immunophenotypic changes were slightly different from previous findings and further large sample size study is warranted to validate these findings. In addition, there were fewer female HBV-HCC patients in our study, which may lead to bias in the results. We need to continue to expand the sample size to reduce gender bias between groups, which will help to discover the characteristics of HBV-HCC circulating immunity in the population and explore whether there are differences in circulating immunity between genders in HBV-HCC. Thirdly, HCC has many causes, including hepatitis B virus, hepatitis C virus, alcohol, etc. In this study, we only described peripheral immunity in patients with HBV-HCC. We need to compare the peripheral immune characteristics of patients with HCC with different causes and to achieve the purpose of individualized treatment for HCC of different etiologies in future. Finally, Single-cell RNA sequencing (scRNA-seq) is a new technique for transcriptome analysis of a large number of single cells. scRNA-seq can provide a deeper understanding of the biological behavior of cells in the complex tumor microenvironment (TME), by analyzing single cell populations (56). Single-cell based transcriptomic analysis has been increasingly used to study the composition of tumor cells and immune cells in normal and disease tissues to better reflect the changes in tumor microenvironment (57). scRNA-seq can provide a comprehensive understanding of the pathogenesis of HCC and is conducive to individualized treatment of HCC. Multiple studies analyzed the scRNA-seq data from healthy controls, tumor tissues and adjacent tissues, and the results showed that the difference of proportion of various immune cells, including CD8^+^ T cells, NK cells, macrophages, B cells and other cells (58–61). The changes of infiltrated immune cells may be related to the immunosuppressive microenvironment in HCC and correlated with HCC progression (62). Previous studies mostly conducted scRNA-seq analysis on HCC tumor tissues and focused on the heterogeneity of tumor microenvironment, while few studies on scRNA-seq of HCC peripheral blood single cells (63). The scRNA-seq analysis of peripheral blood immune cells combined with the immune characteristics of tumor microenvironment would be more beneficial to explore the mechanism of development and the treatment strategy of HCC. Therefore, it is necessary to conduct further research on the scRNA-seq analysis of HBV-HCC peripheral blood immune cells.

## 5 Conclusions

Our study systematically describes the immune landscape of peripheral circulation in HBV-HCC patients and showed that circulating lymphocytes and their subsets in HBV-HCC patients exhibited features of immune exhaustion, especially in HCC patients with persistent viral replication and in patients with advanced HBV-HCC, including decreased frequency of αβ T cells and increased expression of inhibitory receptors including TIGIT and TIM3 on CD4^+^ T cells and γδ T cells, as well as changes in the B cells immune compartment, these changes may be used as potential predictors for evaluating the disease outcome based on the viral replication and disease progression.

Meanwhile, our research suggests that the combination of CD3^+^ T cell and CD8^+^HLADR^+^CD38^+^ T cell may be a potential diagnostic indicator for HBV-HCC. Finally, this study will also provide new evidence for development of novel immunotherapeutic strategies in HBV-HCC patients.

## Data availability statement

The raw data supporting the conclusions of this article will be made available by the authors, without undue reservation.

## Ethics statement

The studies involving human participants were reviewed and approved by The Third Affiliated Hospital of Sun Yat-sen University. The patients/participants provided their written informed consent to participate in this study.Written informed consent was obtained from the individual(s) for the publication of any potentially identifiable images or data included in this article.

## Author contributions

The authors have contributed to the manuscript by proposing the concept, planning the study and revising the manuscript (BW, ZX, YX, ZNY), executing the experiment, analyzing experimental data and writing the manuscript (RNS), collecting patient samples and clinical data (RNS, JWL, XYL, YDY, BL), following up patients and collecting information (RNS, TBL, SX, AYD). All authors contributed to the article and approved the submitted version.

## Supplementary material

Supplementary Table 1 List of fluorescence conjugated antibodies including clone number, Antibody article No and company.
