# Supplementary material for: Peripheral immune characteristics of hepatitis B virus-related hepatocellular carcinoma
Source: Front Immunol. 2023 Apr 3;14:1079495. doi: 10.3389/fimmu.2023.1079495 (PMC10106696; doi:10.3389/fimmu.2023.1079495)
Supplement: Supplementary Table 1 — List of fluorescence conjugated antibodies including clone number, Antibody article No and company. [file Presentation_1.pdf]

Supplementary Figures and Tables

Supplementary Figure 1.

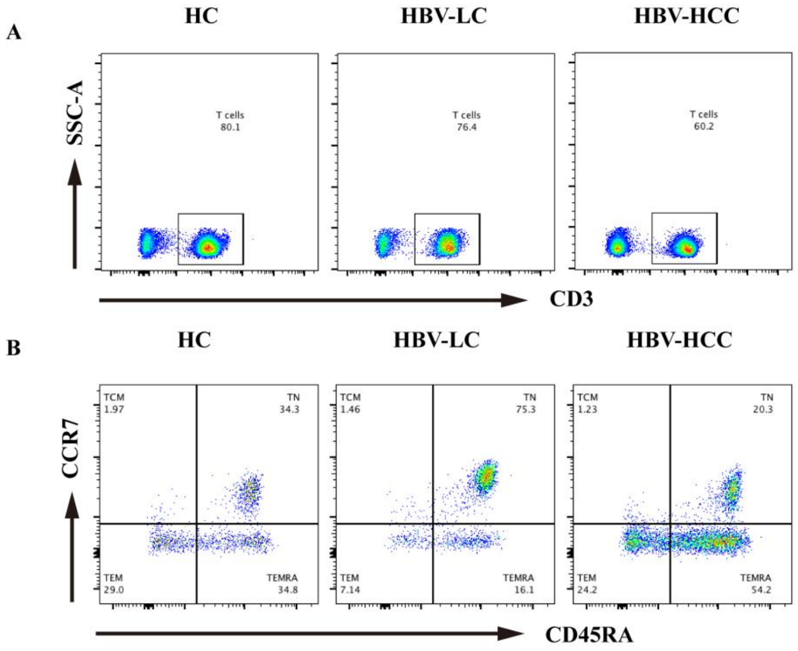

Supplementary Figure 1. The example of flow cytometry scatter plot. (A) The flow cytometry scatter plot of $CD3^+$ T cell. (B) The flow cytometry scatter plot of $CD8^+CCR7^+CD45RA^+$ T cell.

Supplementary Figure 2.

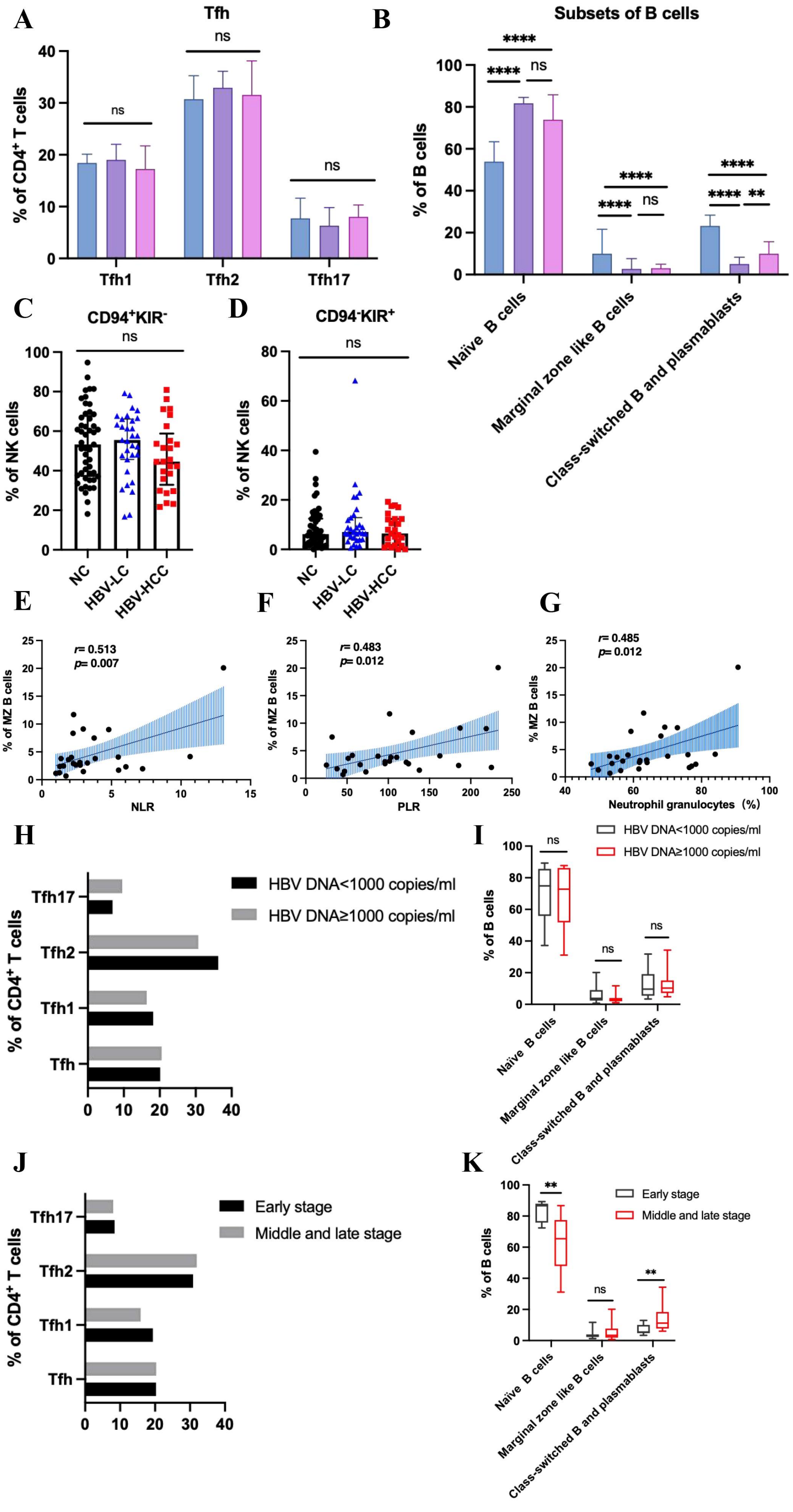
A
Tfh
% of CD4+ T cells
Tfh1
Tfh2
Tfh17
ns
B
Subsets of B cells
% of B cells
Naïve B cells
Marginal zone like B cells
Class-switched B and plasmablasts
****
ns
**
C
CD94+KIR-
% of NK cells
NC
HBV-LC
HBV-HCC
D
CD94-KIR+
E
r= 0.513
p= 0.007
% of MZ B cells
NLR
F
r= 0.483
p= 0.012
PLR
G
r= 0.485
p= 0.012
Neutrophil granulocytes (%)
H
HBV DNA<1000 copies/ml
HBV DNA≥1000 copies/ml
Tfh17
Tfh2
Tfh1
Tfh
I
J
Early stage
Middle and late stage
K

Supplementary Figure 2. (A) The frequency of Tfh1, Tfh2, Tfh17 cells. (B) The frequency of subsets of B cells. (C) The frequency of $CD94^{+}KIR^{-}$ NK cells. (D) The frequency of $CD94^{-}KIR^{+}$ NK cells. (E, F, G) The correlation of MZ B cells and inflammatory markers. (H) The frequency of Tfh, Tfh1, Tfh2, Tfh17 cells between two groups. (I) The frequency of subsets of B cells between two groups. (J) The frequency of subsets of Tfh, Tfh1, Tfh2, Tfh17 cells between two groups. (K) The frequency of subsets of B cells between two groups.

Supplementary Figure 3.

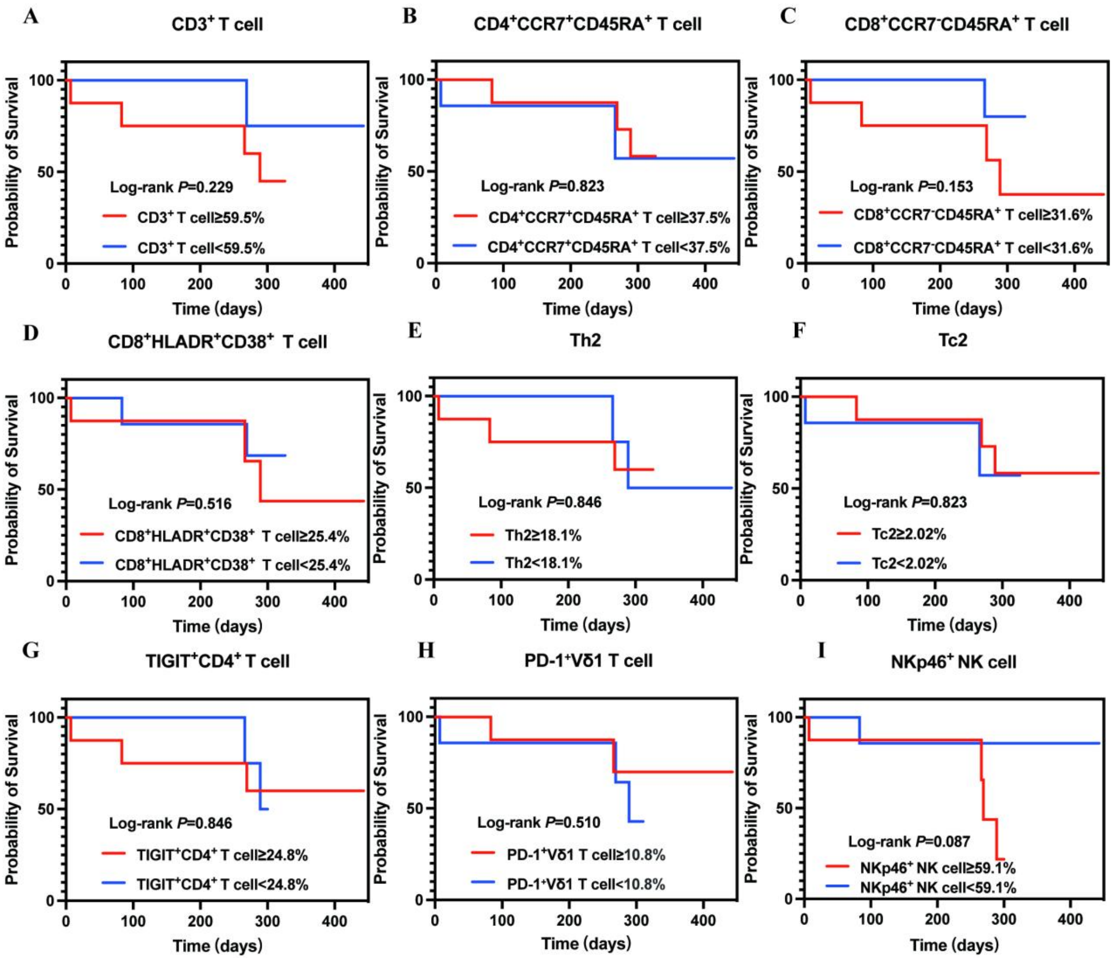

Supplementary Figure 3. Kaplan–Meier curve analysis of immune cells as a predictor of overall survival in HBV-HCC. (A) Kaplan–Meier curve analysis of $CD3^{+}$ T cell. (B) Kaplan–Meier curve analysis of $CD4^{+}CCR7^{+}CD45RA^{+}$ T cell. (C) Kaplan–Meier curve analysis of $CD8^{+}CCR7^{-}CD45RA^{+}$ T cell. (D) Kaplan–Meier

curve analysis of CD8$^+$HLADR$^+$CD38$^+$ T cell. (E) Kaplan–Meier curve analysis of Th2 cell. (F) Kaplan–Meier curve analysis of Tc2 cell. (G) Kaplan–Meier curve analysis of TIGIT$^+$CD4$^+$ T cell. (H) Kaplan–Meier curve analysis of PD-1$^+$ Vδ1 T cell. (I) Kaplan–Meier curve analysis of NKp46$^+$ NK cell.

Supplementary Table 1. List of fluorescence conjugated antibodies including clone number, Antibody article No and company.

Target	Fluorochrome	Clone	Art. No.	Company
CD3	PerCP-Cy5.5	UCHT1	560835	BD
CD3	APC-H7	SK7	560176	BD
CD4	APC-H7	RPA-T4	560158	BD
CD4	PE-Cy7	SK3	557852	BD
CD8	FITC	RPA-T8	301050	Biolegend
CD8	BV510	SK1	563919	BD
CD8	PerCP-Cy5.5	SK1	565310	BD
CD28	PE-Cy7	CD28.2	560684	BD
CD25	PE	M-A251	555432	BD
CD45RA	FITC	HI100	555488	BD
CCR7	Alexa 647	150503	560816	BD
CD127	BV421	HIL-7R-M21	562436	BD
CD57	BV421	NK-1	563896	BD
PD-1	Alexa 647	EH12.1	560838	BD
PD-1	FITC	EH12.2H7	329904	Biolegend
PD-1	BV605	EH12.2H7	329924	Biolegend
HLA-DR	PE	G46-6	555812	BD
CD38	BV510	HIT2	563251	BD
CD38	APC	HIT2	555462	BD
CD45	APC-Cy7	HI30	304014	Biolegend
CD19	PerCP-Cy5.5	HIB19	561295	BD
IgM	BV510	G20-127	563113	BD
IgD	BB515	IA6-2	565243	BD
CD27	BV421	M-T271	562513	BD
CD21	PE-Cy7	B-lY4	561374	BD
CD56	APC	5.1H11	362504	Biolegend
CD94	PerCP-Cy5.5	HP-3D9	562361	BD
KIR/NKB1	PE	DX9	555967	BD
NKG2D	PE-Cy7	1D11	562365	BD
NKP30	Alexa 647	p30-15	325212	Biolegend
NKP30	BV421	p30-15	563385	BD

**NKP46**	**BV510**	**9E2/NKp46**	**564064**	**BD**
**γδ**	**BV421**	**11F2**	**624336**	**BD**
**TCR Vδ1**	**PerCP-vio700**	**REA173**	**130-120-441**	**Miltenyi Biotec**
**TCR Vδ2**	**PE**	**B6**	**555739**	**BD**
**TCR Vδ2**	**BV510**	**B6**	**331432**	**Biolegend**
**LAG3**	**APC-R700**	**T47-530**	**565774**	**BD**
**TIM3**	**APC**	**F38-2E2**	**345012**	**Biolegend**
**TIGIT**	**BV421**	**741182**	**747844**	**BD**
